# Bactericidal Effect of Cecropin A Fused Endolysin on Drug-Resistant Gram-Negative Pathogens

**DOI:** 10.4014/jmb.2205.05009

**Published:** 2022-05-20

**Authors:** Jeonghyun Lim, Juyeon Hong, Yongwon Jung, Jaewon Ha, Hwan Kim, Heejoon Myung, Miryoung Song

**Affiliations:** 1 Department of Bioscience and Biotechnology, Hankuk University of Foreign Studies, Yongin 17035, Republic of Korea; 2 LyseNTech Co., Ltd., Seongnam-si, Gyeonggi-do, 13486, Republic of Korea

**Keywords:** Endolysin, antimicrobial peptide, bacteriophage, gram-negative pathogens, multiple-drug resistance

## Abstract

The rapid spread of superbugs leads to the escalation of infectious diseases, which threatens public health. Endolysins derived from bacteriophages are spotlighted as promising alternative antibiotics against multi-drug resistant bacteria. In this study, we isolated and characterized the novel *Salmonella typhimurium* phage PBST08. Bioinformatics analysis of the PBST08 genome revealed putative endolysin ST01 with a lysozyme-like domain. Since the lytic activity of the purified ST01 was minor, probably owing to the outer membrane, which blocks accessibility to peptidoglycan, antimicrobial peptide cecropin A (CecA) was fused to the N-terminus of ST01 to disrupt the outer membrane. The resulting CecA::ST01 has been shown to have increased bactericidal activity against gram-negative pathogens including *Pseudomonas aeruginosa*, *Klebsiella pneumoniae*, *Acinetobacter baumannii*, *Escherichia coli*, and *Enterobacter cloacae* and the most affected target was *A. baumannii*. In the presence of 0.25 μM CecA::ST01, *A. baumannii* ATCC 17978 strain was completely killed and CCARM 12026 strain was wiped out by 0.5 μM CecA::ST01, which is a clinical isolate of *A. baumannii* and resistant to multiple drugs including carbapenem. Moreover, the larvae of *Galleria mellonella* could be rescued up to 58% or 49% by the administration of CecA::ST01 upon infection by *A. baumannii* 17978 or CCARM 12026 strain. Finally, the antibacterial activity of CecA::ST01 was verified using 31 strains of five gram-negative pathogens by evaluation of minimal inhibitory concentration. Thus, the results indicate that a fusion of antimicrobial peptide to endolysin can enhance antibacterial activity and the spectrum of endolysin where multi-drug resistant gram-negative pathogens can be efficiently controlled.

## Introduction

Development of novel antibiotics relies on the finding of natural compounds and synthetic modification of them but the number of new antibiotics gradually decreased due to a shortage of mined natural compounds [1, 2]. Given this situation, the rapid emergence and spread of resistance to antibiotics resulting from overuse and misuse in both livestock and medical fields have become a major threat to public health in recent years [3, 4]. Moreover, multi-drug resistant, extensively drug-resistant and pandrug-resistant strains are quickly rising, which highlights the urgent need for new treatment options for infectious diseases [5, 6].

Bacteriophages and their endolysins have been considered an alternative agent for the control of bacterial infection. Bacteriophages infect and lyse the host bacteria after replication inside during their life cycle. Many attempts have been made to develop phage therapy as a treatment option for multi-drug resistant bacteria [7–9]. However, it was also reported that bacteria can resist bacteriophage infection through various mechanisms [10–12]. Therefore, more attention has been paid to endolysin, which is a peptidoglycan-degrading enzyme produced by bacteriophages at the end of the lytic cycle to hydrolyze the bacterial cell wall for the release of progeny phage [13]. It has been demonstrated that purified endolysins were found to have strong antibacterial activity against gram-positive pathogens when applied extracellularly [14, 15]. Currently, the first endolysin drug, CF-301, targeting *Staphylococcus aureus*, has entered a phase 3 clinical trial [16]. Endolysin is a promising novel antibiotic alternative since it can act rapidly with no cytotoxicity, and has high efficacy and low probability of resistance development [17].

However, the activity of endolysin is much less effective against gram-negative bacteria due to the outer membrane, which prevents access of endolysin to peptidoglycan. By introducing outer membrane-disrupting peptides or polycationic peptides, the engineered endolysin was expanded to the treatment of gram-negative pathogens [18, 19]. Antimicrobial peptide (AMP) is found in nature and is part of the innate immune system of different organisms with the membrane-disrupting ability [20]. Cecropins were originally isolated from the insect *Hyalophora cecropia* and have antibacterial activity as antimicrobial peptides against gram-negative pathogens [21]. Several studies have reported the increased bactericidal activity of endolysin by the fusion of cecropin A (CecA) at the N-terminus, which works as outer membrane permeabilizing peptides [22, 23].

In this study, we identified the novel endolysin ST01 gene from the genome of the newly isolated *Salmonella typhimurium* phage PBST08, of which protein was shown to have minor antibacterial activity. By fusion with CecA at the N-terminus (CecA::ST01), the lytic activity was improved when it was tested using various gram-negative pathogens including *Acinetobacter baumannii*. The engineered ST01 was effective against not only laboratory strains but also clinical isolates with multiple-drug resistance in vitro and in vivo. Therefore, our results suggest that the introduction of AMP to endolysin can enhance the antibacterial activity and antibacterial spectrum of endolysin.

## Materials and Method

### Bacterial Strains and Growth Conditions

The bacterial strains used in this study were purchased from the American Type Culture Collection (ATCC), the Korean Collection for Type Cultures (KCTC), and the Culture Collection of Antimicrobial Resistance Microbes (CCARM). F strains were generous gifts from Professor Kwan Soo Ko (Sungkyunkwan University). *E. coli* DH5α was used for DNA cloning and BL21(DE3) pLysS was used for protein overexpression. All bacterial strains were grown in Luria Bertani (LB; MBcell, Korea) broth at 37°C with vigorous aeration unless otherwise noted. Antibiotics and chemicals were added when necessary: ampicillin (100 μg/ml), chloramphenicol (30 μg/ml) and isopropyl-B-D-thiogalactopyranoside (IPTG) at indicated concentrations.

### Genome analysis of Bacteriophage PBST08 and Bioinformatic Analysis of Endolysin ST01

The bacteriophage was isolated from Yeongsan River, Jeollanam-do, Korea by the soft agar overlay method [24]. Briefly, a single plaque was selected from the overlaid LB top agar (final 0.7 %) containing a mixture of wastewater and cultured *Salmonella typhimurium* ATCC 14028 at log phase (OD_600_ = 0.5) after overnight incubation at 37°C. Phages were then purified by centrifugation of the plaque in 10% (w/v) polyethylene glycol 8000 (PEG 8000) [25] and named PBST08. Whole genome sequences were identified by Illumina Miseq (LAS, Korea) using purified genomic DNA with the Phage DNA isolation kit (Norgen #46800, Canada). Genome assembly and annotation was performed by SAVAGE, followed by remapping to sequence reads and manual curation. By BLAST analysis, open reading frames (ORFs) were predicted and putative endolysin ST01 was identified by comparison analysis using the SwissProt and Pfam databases. The amino acid sequence of ST01 was aligned using the ESPript 3.0 server [26] and ClustalW [27]. Domain analysis was performed with the InterPro tool [28].

### Endolysin Overexpression and Purification

The ORF114 encoding endolysin ST01 was amplified using the following primers: forward 5’-atgcGGATCCATGTCAAACCGAAACATTAG-3’ and reverse 5’-aataaCTCGAGCTTTGCTTCGCGGCCGATG-3’. The amplicon was cleaned up with the MinElute^®^ Reaction Cleanup Kit (Qiagen, Germany) and digested with BamH1 (NEB, USA) and Xho1 (NEB, USA). The DNA fragment was then ligated with pET21a and the resulting plasmid pAS008 was verified by sequencing analysis. The plasmid containing antimicrobial peptide cecropinA (CecA) fused ST01 was constructed by Gibson assembly of the amplified ST01 fragment and pET21a with CecA. The ST01 fragment was amplified with pAS008 using froward primer 5’-CAGCGGCTCGGGTAGTATGTCAAA CCGAAACATTAGTAACAACGGC -3’ and reverse primer 5’-GCAGCAGCCAACTCAGCTT-3’. The pET21a with CecA was prepared by PCR using LNT113 [23] as a template and primers: froward primer 5’-CTAACA AAGCCCGAAAGGAAGCTG -3’ and reverse primer 5’-GCCGTTGTTACTAATGTTTCGGTTTGACATACT ACCCGAGCCGCTG -3’. The assembled plasmid containing CecA::ST01 was named pAS047. The plasmid pAS008 or pAS047 was introduced into BL21 (DE3) pLysS strain for the purification of ST01 or CecA::ST01, respectively.

BL21 (DE3) pLysS carrying pAS008 or pAS047 was grown in 1.5 L of LB broth containing ampicillin and chloramphenicol at 37°C. When optical density at 600 nm (OD_600_) reached 0.6, IPTG (1 mM) was added for the induction of the recombinant protein and further incubated at 37°C for 5 h for ST01 or at 18°C for 21 h for CecA::ST01. The cells were harvested by centrifugation at 3,500 ×*g* at 4°C for 10 min and the cell pellet was resuspended in 100 ml of lysis buffer [20 mM Tris-HCl pH 7.5, 0.5 M NaCl, 10 mM imidazole], followed by cell disruption using a sonicator with 50% maximum amplitude for 10 min with 5-s pulse/ 15-s break steps on ice. The unbroken cells were separated by centrifugation at 14,000 ×*g* at 4°C for 30 min and the supernatant was filtered through a 0.45 μm-pore-size filter (GVS, Italy). The filtered fraction was then applied to 5 ml of an HisTrap HP column (Cytiva, USA) connected to an AKTA go fast protein liquid chromatography (FPLC) system controlled by Unicorn 5.1 software. The bound endolysins were eluted using a buffer [20 mM Tris-HCl pH 7.5, 0.5 M NaCl] with a gradient of imidazole concentration from 10 mM to 0.5 M and the eluted fractions containing endolysins were further applied into a 5 ml of an HiTrap SP column (Cytiva). The proteins were eluted with a second buffer [20 mM Tris-HCl pH 7.5, 1 M NaCl] and dialyzed overnight in 1×PBS at 4°C with stirring. The protein concentration was measured with a the Bradford assay kit using bovine serum albumin (BSA) as a standard (Bio-Rad, USA).

### Plate Lysis Assay

Overnight culture of *S. typhimurium* ATCC 14028 was harvested by centrifugation at 3,200 ×*g* for 10 min. The pellet was resuspended in 1 ml LB and autoclaved at 121°C for 15 min. The top agar mixture with autoclaved bacteria and Triton X-100 (0.1%) was overlaid on an LB plate. SoluBL21 carrying pAS008 was grown in fresh LB broth containing ampicillin at 37°C until OD_600_ reached 0.6. After the addition of 0 or 1 mM IPTG for the induction of ST01 expression, cells were further grown at 37°C for 6 h. SoluBL21 strain with pET21a was used as a control strain for the assay and prepared as a sample strain except for the addition of IPTG. One microliter of each strain was spotted on the top agar overlaid plate and incubated at 37°C overnight.

### CFU Reduction Assay

The antibacterial activity of ST01 or CecA::ST01 was determined using *Salmonella typhimurium*, *Pseudomonas aeruginosa*, *Escherichia coli*, *Acinetobacter baumannii*, *Klebsiella pneumoniae*, *Enterobacter aerogenes*, and *Enterobacter cloacae*. Bacterial cells at the mid-exponential phase (OD_600_ =0.8) were harvested by centrifugation at 15,000 ×*g* for 1 min and adjusted to 1 × 10^6^ CFU/ml in 20 mM Tris-HCl pH 7.5 after washing. The bacterial samples were mixed with the indicated concentration of the purified ST01 or CecA::ST01. After 2 h incubation at 37°C, cells were serially diluted in 1×PBS and spotted on LB agar for the enumeration of the surviving bacteria.

### *Galleria mellonlella* Infection Models

Healthy *Galleria mellonella* larvae from Sworm (Cheonan, Korea) was selected to weigh from 30 to 130 mg in the final instar stage. The larvae were deprived of food and stored at 30°C for one day before bacterial infection. Exponentially grown *A. baumannii* ATCC 17978 or CCARM 12026 strain was harvested by centrifugation at 3,500 ×*g* for 5 min and resuspended in 1× PBS. The larvae were then injected with 5 μl of the mixture containing 5μM of CecA::ST01 and 1×10^6^ CFU of each strain, which was prepared immediately before injection. All injections were delivered into the last left proleg using a 10R-GT 10 μl syringe (Trajan Scientific and Medical, Australia; *n* = 10). The infected larvae were incubated at 30°C for 72 h and survival was monitored by checking melanization and response to touch. As a control, a bacterial mixture with 1xPBS was injected into larvae (mock; *n* = 10). The experiment was repeated at least three times.

### Measurement of Minimal Inhibitory Concentration (MIC)

The MIC values of CecA::ST01 were determined by broth microdilution method in 96-well plates as described in [23] with the strains of *P. aeruginosa*, *K. pneumoniae*, *A. baumannii*, *E. coli*, and *E. cloacae*. Each strain grown in LB overnight was inoculated and cultured in CAA medium (5 g/l casamino acids, 5.2 mM K_2_HPO_4_, and 1 mM MgSO_4_) at 37°C for 3.5 h. The cells were diluted to 1 × 10^4^ CFU/ well in CAA medium and incubated with the purified CecA::ST01 (1-128 μg/ml) at 37°C for 20 h. The MIC was determined as the lowest concentration for inhibiting bacterial growth.

### Statistical Analysis

Data was analyzed using GraphPad Prism software version 9.3.0. A two-tailed Student’s t-test was used for the analysis of the differences between each dataset and a log-rank (Mantel-Cox) test was used for survival experiments. All data were presented as mean ± SD, and differences were considered significant at *p* <0.05.

## Results

### Identification of Novel Endolysin ST01 from *Salmonella typhimurium* Phage PBST08

**Fig. 1:**
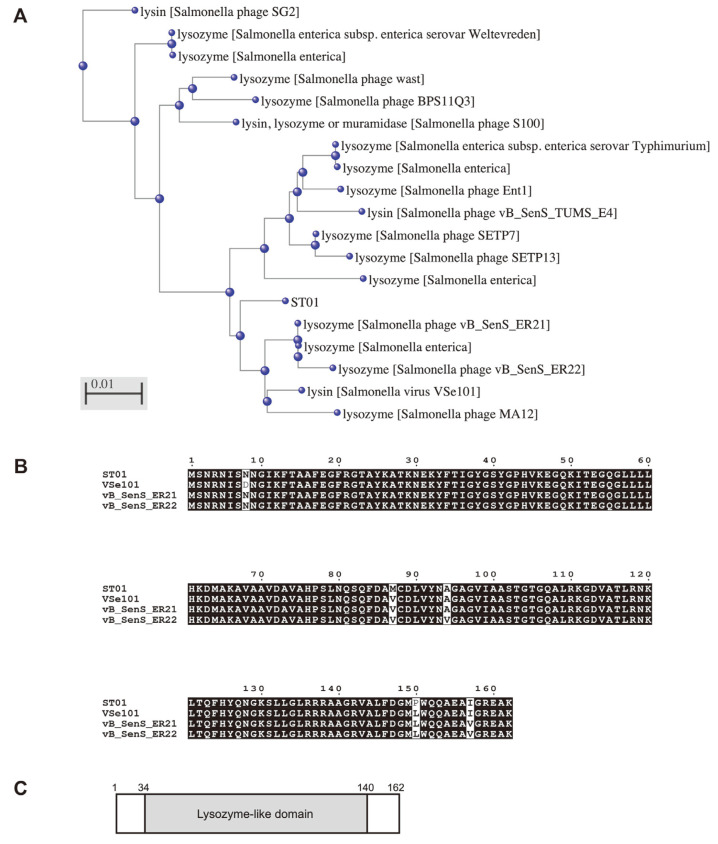
Identification of novel phage endolysin ST01. (**A**) Cladogram of ST01 with 18 proteins using the Constraint-based Multiple Alignment Tool (COBALT). (**B**) Multiple alignment analysis of amino acid sequence of ST01 with *Salmonella* phage vB_SenS_ER21 lysozyme, *Salmonella* virus VSe101 lysin, and *Salmonella* phage vB_SenS_ER22 lysozyme. The alignment was performed using ClustalW. Black boxes represent 100% sequence identity. (**C**) The domain structure of ST01 was predicted by the InterPro tool. The predicted lysozyme-like domain is located between amino acid 34 and 140 of ST01.

A putative endolysin was predicted from the newly isolated bacteriophage PBST08 using *Salmonella typhimurium* ATCC 14028 as a host. The genomic sequence of phage PBST08 had 98.66% identity to *Salmonella phage* TS3 with 99% query coverage by BlastN analysis (Table S1). Based on NCBI ORF finder analysis, a total of 208 ORFs were identified and 45 functional ORFs were clustered into four functional groups structural protein, replication and regulation, DNA packaging, and lysis (Fig. S1, Table S2). Among them, five ORFs were related to lysis and ORF114 was annotated as a putative endolysin, named ST01. The ST01 endolysin was similar to 18 proteins with over 96%identity, the function of which are known as lysin or lysozyme from various *Salmonella* phages (Fig. 1A). BlastP analysis revealed that the amino acid sequence of ST01 is 98.15% identical to the lysozyme of *Salmonella* phage vB_SenS_ER21, lysin of *Salmonella* virus VSe101, and 98.11% identical to the lysozyme of *Salmonella* phage vB_SenS_ER22 (Fig. 1B). And, the ST01 has a lysozyme-like domain based on functional domain analysis (Fig. 1C).

**Fig. 2:**
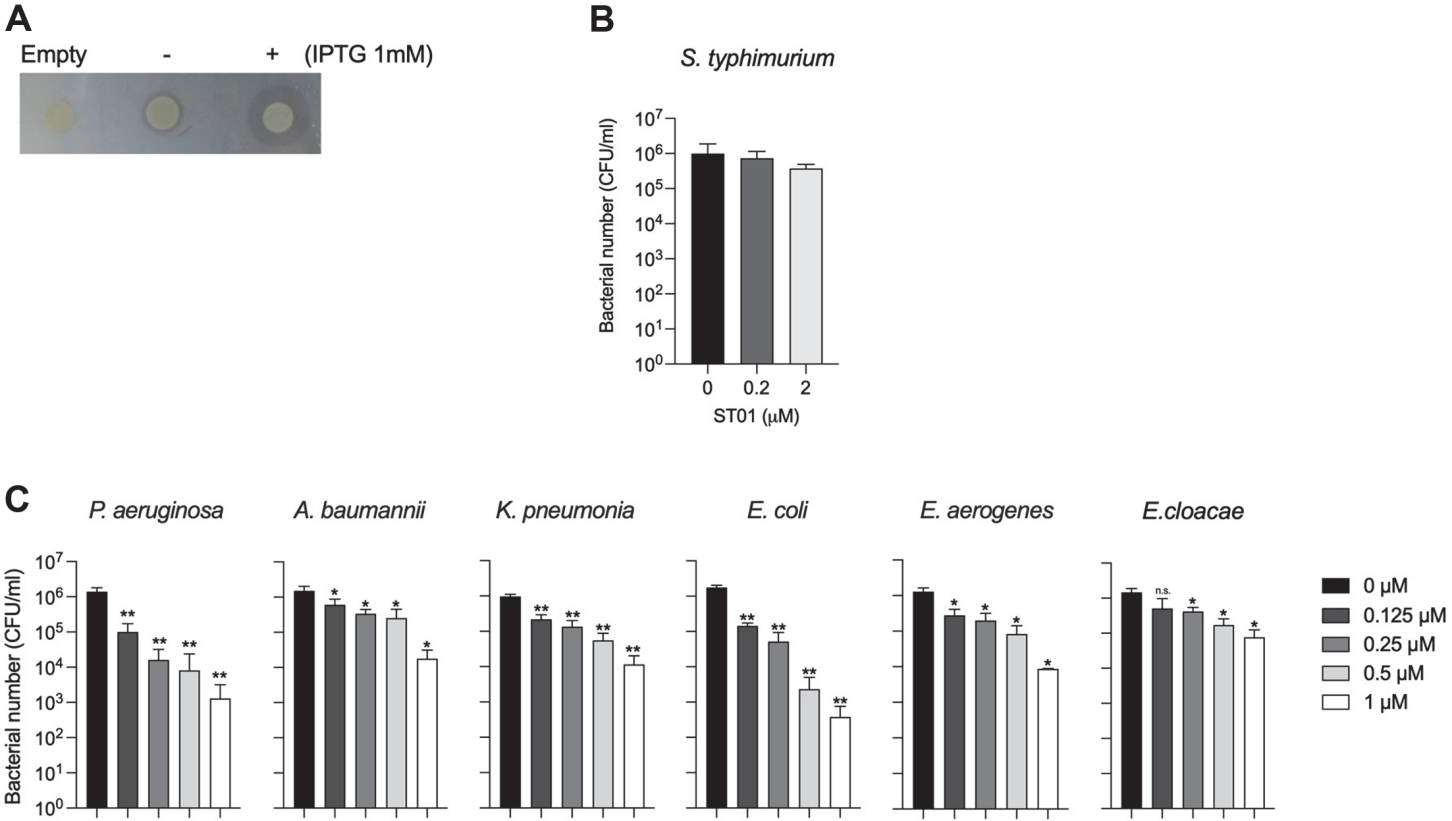
Lytic activity of ST01 against *S. typhimurium*. (**A**) The peptidoglycan-degrading activity of ST01 was determined by plate lysis assay using a plate containing an autoclaved culture of *Salmonella typhimurium* ATCC 14028. SoluBL21 carrying pET21a (Empty) or pAS008 was grown in LB containing ampicillin until culture reached the midexponential phase and expression of ST01 was induced with the addition of 0 mM (-) or 1 mM IPTG (+). After 6 h culture at 37°C, 1 μl of each culture was spotted on the plate and air dried. The plates were incubated at 37°C overnight. The clear zone represents the lytic activity of ST01. (**B**) The antimicrobial activity of ST01 against *S. typhimurium* ATCC 14028 was tested by CFU reduction assay. Exponentially grown bacterial cells were adjusted as 1 × 10^6^ CFU in 20 mM Tris-HCl pH 7.5 and treated with 0, 0.2, and 2 μM of purified ST01 at 37°C for 2 h. The surviving bacterial cells were counted by plating on an LB plate. (**C**) The antimicrobial activity of ST01 was tested against *P. aeruginosa* PA01, A.baumannii ATCC 17978, *K. pneumonia* KCTC 2208, *E. coli* ATCC 8739, *E. aerogenes* F276, *E. cloacae* ATCC 13047 by CFU reduction assay. The bacterial cells were prepared as described above and treated with 0, 0.125, 0.25, 0.5, 1 μM of ST01. The experiments were repeated at least three times and data are presented as mean ± SD. Significance is shown as *p < 0.0392; **p < 0.0074; n.s. = not significant.

To verify the predicted function of ST01, ORF114 was cloned into pET21a and the resulting plasmid pAS008 was then transformed into BL21 (DE3) strain. The BL21(DE3) strain carrying pAS008 was tested for endolysin activity by plate lysis assay using the autoclaved culture of *S. typhimurium* (Fig. 2A). A halo zone was observed around the strain expressing ST01with the addition of 1 mM IPTG (+; right), but not around the bacterial strain with pET21a (Empty; left). There was also a thin clear zone around the strain with pAS008 without induction (–; middle), which is probably due to leakage expression. Thus, the results indicate that ST01 has lytic activity, degrading peptidoglycan, as predicted by bioinformatic analysis. Next, ST01 protein purified from SoluBL21 was evaluated for lytic activity against live *S. typhimurium* cells by CFU reduction assay (Fig. 2B). Although ST01 was from the *Salmonella* phage, the lytic activity of the purified ST01 was marginal against *Salmonella* when it was applied outside of cells. The cell number of *Salmonella* decreased by less than one log with the treatment of 2 μM ST01 and the endolysin activity of ST01 was more effective against *P. aeruginosa*, *K. pneumoniae*, *A. baumannii*, *E. coli*, and *E. cloacae*, the numbers of which decreased up to 4 logs with 1 μM of ST01 (Fig. 2C). Therefore, it was confirmed that ST01 indeed has lytic activity as predicted as an endolysin.

### Engineering of ST01 by a Fusion with Cecropin A

**Fig. 3:**
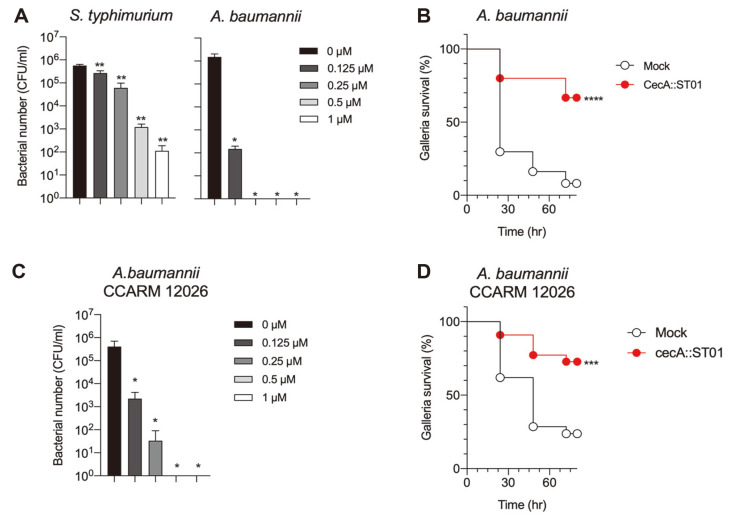
Evaluation of lytic activity of CecA::ST01 against *A. baumannii* in vitro and in vivo. (**A**). The antibacterial activity of CecA::ST01 was determined using *S. typhimurium* ATCC 14028 and *A. baumannii* ATCC 17978. Each bacterial suspension in 20 mM Tris-HCl pH 7.5 was treated with 0, 0.125, 0.25, 0.5, and 1 μM of CecA::ST01. Significance is indicated as **P<0.006; *P<0.0106. Data are presented as mean ± SD (*n* = 3) (**B**). Survival rates of *A. baumannii* ATCC 17978 infected larvae of *G. mellonella* treated with no (mock) or 5 μM of CecA::ST01. The larvae were infected by 2 × 10^6^ CFU of *A. baumannii* ATCC 17978. For endolysin treatment, 5 μM of CecA::ST01 was mixed with bacterial suspension immediately before injection. The survival of larvae was monitored by the time the larvae were kept at 30°C for 72 h (*n* = 10 per group). The experiment was repeated three times. Significance is shown as ****p < 0.0001. (**C**) The lytic activity of CecA::ST01 was determined using the clinical isolate of *A. baumannii*, CCARM 12026 by CFU reduction assay. Significance is indicated as *p < 0.0201. Data are presented as mean ± SD (*n* = 3) (**D**). The larvae of *G. mellonella* was infected by 2 × 10^6^ CFU of *A. baumannii* CCARM 12026. For endolysin treatment, 5 μM of CecA::ST01 was mixed with bacterial suspension immediately before injection. As a control, the same amount of 1×PBS was used (mock). The survival of larvae was monitored by the time the larvae were kept at 37°C for 72 h (*n* = 10 per group). The experiment was repeated three times. Significance is shown as ***p < 0.0009.

Although the endolysin activity of ST01 was verified, the lytic activity against gram-negative pathogens was low. Since the outer membrane of gram-negative bacteria blocks the interaction between the peptidoglycan substate and endolysin, membrane-destabilizing peptide cecropin A (CecA) was fused to the N-terminus of ST01, CecA::ST01, to improve antibacterial activity. Of note, CecA itself could decrease the bacterial number up to 2 logs and CecA fused EGFP did not show any antibacterial activity in our previous study [23]. The purified CecA::ST01 was analyzed by SDS-PAGE and degradation or impurity was not detected (Fig. S2). As shown in Fig. 3A, the bacteriolytic activity of ST01 was dramatically enhanced by the introduction of CecA when it was applied to *Salmonella*. More than 4 logs of *Salmonella* was killed with 1 μM of CecA::ST01. Moreover, CecA::ST01 could eliminate all *A. baumannii* cells when it was treated with more than 0.25 μM. Other gram-negative pathogens were also efficiently removed by CecA::ST01, where *P. aeruginosa* PA01, *K. pneumonia* KCTC 2208, and *E. coli* ATCC 8739 were completely killed and 2~3 log of *E. aerogenes* F276 and *E. cloacae* ATCC 13047 were reduced by treatment with 1 μM of CecA::ST01 (Fig. S3). Additionally, the biochemical analysis showed that the lytic activity of CecA::ST01 was not affected by changes in pH, salt concentration and temperature (Fig. S4), indicating the superb stability of CecA::ST01. Next, the antibacterial activity of CecA::ST01 was further evaluated in vivo using a *Galleria mellonella* infection model with *A. baumannii* (Fig. 3B). Only 10% of infected *G. mellonella* survived with no endolysin treatment (mock). Approximately 58% of the infected worms survived when CecA::ST01 was administered to the larvae, which was mixed with bacterial suspension immediately before injection with no bactericidal effects (data not shown). Altogether, the antibacterial activity of endolysin ST01 against gram-negative pathogen was improved with the addition of the outer membrane-disrupting peptide CecA, which was verified in vitro and in vivo.

### Control of Multi-Drug Resistant *A. baumannii* Infection by CecA::ST01

Since the potential of CecA::ST01 as an alternative antibiotic was validated using laboratory strain, the antibacterial activity was further investigated using the clinical isolate of *A. baumannii* CCARM 12026 strain with multi-drug resistance (MDR) including amikacin, ceftazidime, ciprofloxacin, cefotaxime, gentamicin, piperacillin and tobramycin. First, CecA::ST01 was used for the measurement of lytic activity with the culture of CCARM 12026 strain in vitro. The number of CCARM 12026 gradually decreased with the treatment with increased concentrations of CecA::ST01 in the CFU reduction assay (Fig. 3C). When CecA::ST01 was treated at more than 0.5 μM, *A. baumannii* CCARM 12026 was completely eradicated. Finally, the in vivo efficacy of CecA::ST01 was tested using a *G. mellonella* infection model with *A. baumannii* CCARM 12026 strain (Fig. 3D). It was as effective as against ATCC 17978, and CecA::ST01 increased the survival of infected larvae with MDR *A. baumannii* by up to 49%. Therefore, the engineered endolysin CecA::ST01 can be used for the control of infection by the MDR *A. baumannii* strain.

### Antibacterial Spectrum of CecA::ST01 against Gram-Negative Pathogens

Since the efficacy was validated against the representative gram-negative pathogen *A. baumannii*, the antibacterial spectrum of CecA::ST01 was further estimated against various gram-negative pathogens including drug-resistant strains and clinical isolates of *P. aeruginosa*, *K. pneumoniae*, *A. baumannii*, *E. coli*, and *E. cloacae* by determination of MIC (Table 1). CecA::ST01 did not effectively eliminate the strains of *P. aeruginosa*, where MICs were higher than 32 μg/ml for two strains or could not be determined (higher than 128 μg/ml) for four strains. But most strains were killed by CecA::ST01 regardless of antibiotic resistance with MICs ranging from 4 to 64 μg/ml. In particular, the strains of *A. baumannii* and *E. coli* were highly susceptible to CecA::ST01 with MICs less than 8 μg/ml. Collectively, the results indicate that CecA::ST01 can be a practical agent against gram-negative pathogens including MDR strains.

## Discussion

Bacteriophage-encoded endolysin is a promising antibacterial with high specificity and low resistance potential that degrades the peptidoglycan of the cell wall with enzymatic activity [29]. However, accessibility to its substrate, peptidoglycan, restricts the enzymatic activity of endolysin in the case of gram-negative bacteria protected by a thick outer membrane [30]. By engineering natural endolysin, the endolysin could penetrate the outer membrane and then degrade its substrate where the fused peptide can disrupt the major component of the outer membrane such as lipopolysaccharide [18, 31, 32]. Therefore, mining new endolysins followed by intensive engineering can provide new treatments for infection by drug-resistant gram-negative pathogens. In an attempt to develop a newly engineered endolysin, we first identified a novel endolysin gene with a lysozyme-like domain, ST01, by bioinformatic analysis of the genome of the new *S. typhimurium* bacteriophage PBST08. The purified ST01 was applied to the culture of various gram-negative pathogens including native host *S. typhimurium*, but antibacterial activity was not distinctive, which suggests that endolysin ST01 should be engineered to overcome the barrier, the outer membrane. Next, we introduced the antimicrobial peptide cecropinA (CecA) at the N-terminus of ST01, since CecA was shown to be effective in improving the efficacy of another endolysin in a previous study [23]. The resulting endolysin, CecA::ST01, could eliminate all tested strains except *Enterobacter* spp., and *A. baumannii* was the most susceptible strain. Also, the activity of CecA::ST01 was well maintained upon stresses induced by high osmolarity, shifting pH and thermal changes. The results indicate that CecA::ST01 has strong antibacterial activity with a broad spectrum and high stability. When in vivo efficacy was tested using a *G. mellonella* infection model with *A. baumannii*, the survival of larvae increased up to 58 % by treatment with CecA::ST01. Most interestingly, MDR strains of *A. baumannii* were effectively killed by CecA::ST01 in vitro, thus guaranteeing the survival of the infected larvae. Thus, CecA::ST01 is an effective antibacterial agent for many strains of gram-negative pathogens but, most strains of *P. aeruginosa* were not efficiently killed by CecA::ST01. On the other hand, other CecA fused endolysins such as LNT113 was successfully control the various strains of *P. aeruginosa* [23]. The antibacterial activity of engineered endolysin is dependent on the biochemical characteristics of endolysin, which is an enzyme with specificity for its substrate and each bacteria has subtle differences in the composition and structure of peptidoglycan. Therefore, each CecA fused endolysin can have differences in the target spectrum, but this can be resolved by further protein engineering. This study proves that engineered endolysin containing an antimicrobial peptide can be applied to the control of infection by gram-negative pathogens regardless of drug resistance with high efficacy and stability. In a future study, the efficacy of an engineered endolysin should be addressed in an animal model to evaluate the feasibility of the protein as an alternative to antibiotics in humans.

## Supplemental Materials

Supplementary data for this paper are available on-line only at http://jmb.or.kr.

## Figures and Tables

**Table 1:** MIC values of CecA::ST01 against gram-negative pathogens.

Bacterial strains	MIC (μg/ml)	Bacterial strains	MIC (μg/ml)
*P. aeruginosa*		*E. cloacae*	
PAO1	32	ATCC 13047	8
ATCC 13388	>128	CCARM 16012	64
ATCC 9027	>128	CCARM 16014	16
ATCC 27853	32	CCARM 16017	16
F341	>128	CCARM 0252	4
CCARM 2092	>128	CCARM 16003	4
CCARM 2134	64		
*K. pneumoniae*		*A. baumannii*	
KCTC 2208	4	ATCC 19606	4
ATCC 700603	16	ATCC 17978	4
CCARM 10143	16	F66	4
CCARM 10225	16	F68	4
CCARM 10236	16	CCARM 12026	8
CCARM 10269	8	CCARM 12035	8
*E. coli*			
ATCC 8739	8		
ATCC 25922	4		
F906	8		
CCARM 1A746	4		
CCARM 1B684	4		
CCARM 1460	4		
