# Supplementary material for: Bactericidal Effect of Cecropin A Fused Endolysin on Drug-Resistant Gram-Negative Pathogens
Source: J Microbiol Biotechnol. 2022 May 20;32(6):816–23. doi: 10.4014/jmb.2205.05009 (PMC9628910; doi:10.4014/jmb.2205.05009)
Supplement: Supplementary file 1 [file jmb-32-6-816-supple.pdf]

https://doi.org/10.4014/jmb.2205.05009

Table S1. Comparison of genome sequences of phage PBST08 by BLAST analysis.

Description	Query cover	Per. Ident	Accession No.
Salmonella phage TS3, complete genome	99%	98.66%	MK249126.1
Salmonella phage MA12, complete genome	99%	98.96%	KX245013.1
Salmonella phage ST1, partial genome	99%	98.57%	MF001366.1
Salmonella phage ST3, partial genome	99%	98.84%	MF001364.1
Salmonella phage PIZ SAE-01E2, complete genome	96%	91.61%	MN336266.1
Salmonella phage vB_SenS_ER22, complete genome	95%	92.28%	MW355464.1
Salmonella phage vB_SenS_ER23, complete genome	95%	92.49%	MW355465.1
Salmonella phage vB_SenS_ER21, complete genome	95%	91.39%	MW355463.1
Salmonella phage f18SE, complete genome	94%	91.01%	KR270151.1

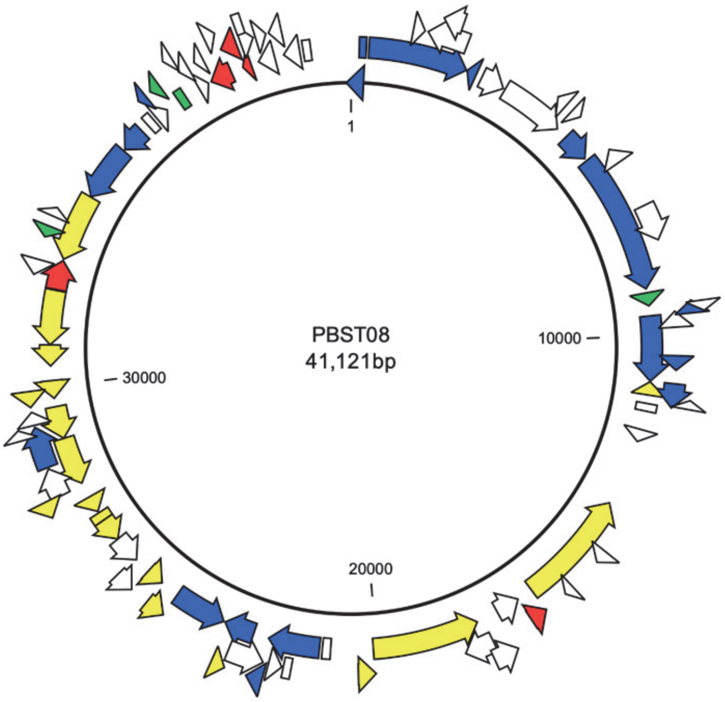

Figure S1. Open reading frame (ORF) map of PBST08 phage. Open reading frame (ORF) was analyzed using NCBI BLAST and map was drawn using CLC workbench 20. Each arrow represents a functionally annotated ORF and is colored according to its function. Red, lysis; yellow, structural protein; green, DNA packaging; blue, replication and regulation; white, uncharacterized protein.

Table S3. Functional annotation of open reading frames (ORFs) of phage PBST08

Predicted function	ORF number	Description	Related bacteriophage	Coverage (%)	Identity (%)
Structural protein	ORF122	o-spanin [Salmonella phage PIZ SAE-01E2]	Salmonella phage PIZ SAE-01E2	95%	99.19%
	ORF123	head outer capsid protein [Escherichia phage buks]	Escherichia phage buks	95%	96.55%
	ORF124	head-tail joining protein [Salmonella phage STP03]	Salmonella phage STP03	100%	98.39%
	ORF125	head-tail joining protein [Salmonella phage sidste]	Salmonella phage sidste	100%	94.67%
	ORF126	HK97 gp10 family phage protein [Salmonella enterica]	Salmonella enterica	100%	100.00%
	ORF132	tail fiber protein [Salmonella phage TS3]	Salmonella phage TS3	100%	99.77%
	ORF133	tailspike protein [Salmonella phage L13]	Salmonella phage L13	100%	99.85%
	ORF153	tail protein [Salmonella phage L13]	Salmonella phage L13	100%	99.34%
	ORF154	o-spanin [Salmonella phage BPS11Q3]	Salmonella phage BPS11Q3	81%	85.06%
	ORF155	scaffold protein [Salmonella phage fmb-p1]	Salmonella phage fmb-p1	99%	98.28%
	ORF156	capsid protein [Salmonella phage MA12]	Salmonella phage MA12	100%	100.00%
	ORF157	capsid and scaffold protein [Salmonella phage SE-W109]	Salmonella phage SE-W109	86%	97.87%
	ORF163	tail assembly chaperone [Escherichia phage vB_EcoS_XY1]	Escherichia phage vB_EcoS_XY1	100%	84.03%
	ORF188	structural protein [Salmonella phage LPSE1]	Salmonella phage LPSE1	100%	98.98%
	ORF190	head morphogenesis protein [Salmonella phage 5sent1]	Salmonella phage 5sent1	90%	95.80%
	ORF193	tail protein [Salmonella phage sidste]	Salmonella phage sidste	100%	96.40%
	ORF194	tail assembly chaperone [Salmonella phage vB_SenS-EnJE1]	Salmonella phage vB_SenS-EnJE1	100%	97.83%
	ORF196	tape measure protein [Salmonella phage BPS11Q3]	Salmonella phage BPS11Q3	99%	98.20%
Replication and regulation	ORF2	DNA polymerase [Salmonella phage BPS11Q3]	Salmonella phage BPS11Q3	100%	99.52%
	ORF3	DNA polymerase I [Salmonella phage MA12]	Salmonella phage MA12	100%	99.03%
	ORF5	DNA helicase [Salmonella phage SE40]	Salmonella phage SE40	72%	52.86%

	ORF34	replicative DNA helicase/primase [Salmonella phage TS3]	Salmonella phage TS3	100%	99.86%
	ORF42	HNH endonuclease [Salmonella phage L13]	Salmonella phage L13	100%	100%
	ORF53	phosphodiesterase [Escherichia phage Shashou]	Escherichia phage Shashou	70%	89.33%
	ORF54	putative DNA-binding protein [Salmonella phage L13]	Salmonella phage L13	100%	100%
	ORF75	replication protein [Escherichia phage vB_EcoS_XY1]	Escherichia phage vB_EcoS_XY1	100%	67%
	ORF82	DEAD/DEAH box helicase [Salmonella enterica]	Salmonella enterica	100%	99%
	ORF90	metallophosphoesterase [Salmonella enterica]	Salmonella enterica	100%	100%
	ORF99	putative helicase [Salmonella phage fmb-p1]	Salmonella phage fmb-p1	59%	92%
	ORF140	helix-turn-helix domain-containing protein [Salmonella enterica]	Salmonella enterica	100%	100%
	ORF151	DNA-binding transcriptional regulator [Salmonella phage 5sent1]	Salmonella phage 5sent1	96%	88%
	ORF152	terminase small subunit [Salmonella phage dunkel]	Salmonella phage dunkel	99%	99%
	ORF160	site-specific recombinase [Salmonella phage ST3]	Salmonella phage ST3	100%	99%
	ORF171	putative intein containing helicase [Salmonella phage fmb-p1]	Salmonella phage fmb-p1	92%	88%
	ORF180	DNA-binding protein [Raoultella phage RP180]	Raoultella phage RP180	33%	77%
	ORF187	terminase large subunit [Salmonella phage ST3]	Salmonella phage ST3	100%	100%
Lysis	ORF62	amidase [Salmonella phage ST4]	Salmonella phage ST4	100%	96.76%
	ORF114	lysozyme [Salmonella phage vB_SenS_ER21]	Salmonella phage vB_SenS_ER21	100%	98.15%
	ORF147	putative holin [Salmonella phage Jersey]	Salmonella phage Jersey	100%	95.24%
	ORF164	peptidoglycan endopeptidase [Escherichia phage phiWAO78-1]	Escherichia phage phiWAO78-1	100%	76.86%
	ORF184	holin class I [Salmonella phage Shelanagig]	Salmonella phage Shelanagig	85%	94.74%
DNA packaging	ORF81	restriction endonuclease [Salmonella phage BPS11Q3]	Salmonella phage BPS11Q3	100%	95.79%
	ORF116	NinH-like protein [Salmonella virus VSe103]	Salmonella virus VSe103	100%	98.65%
	ORF119	portal protein [Salmonella phage templet]	Salmonella phage templet	63%	63.27%
	ORF185	Nin protein [Salmonella phage ST3]	Salmonella phage ST3	100%	100.00%

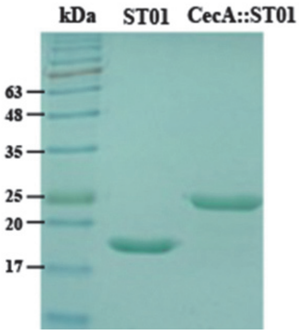

Figure S2. Analysis of purified ST01 and CecA::ST01. SDS-PAGE analysis of the purified endolysins, which were purified by Fast Protein Liquid Chromatography (FPLC) using His column and SP column.

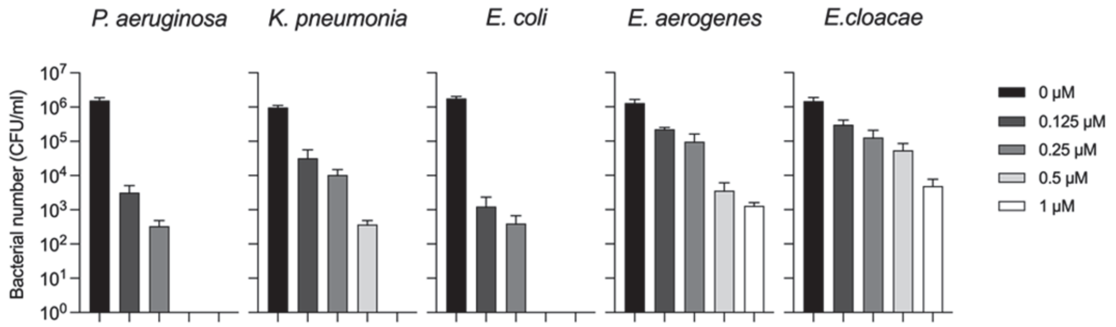

Figure S3. Antibacterial activity of CecA::ST01 against gram-negative pathogens. Antimicrobial activity of CecA:: ST01was tested by CFU reduction assay using *P. aeruginosa* PA01, *A.baumannii* ATCC 17978, *K. pneumonia* KCTC 2208, *E. coli* ATCC 8739, *E. aerogenes* F276, *E. cloacae* ATCC 13047. Bacterial cells at the mid-exponential phase (OD_{600}=0.8) were harvested by centrifugation at 15,000 x g for 1 min and adjusted to $1x10^6$ CFU/ml in 20 mM Tris-HCl pH 7.5 after washing, followed by the treatment with 0, 0.125, 0.25, 0.5, 1 μM of CecA::ST01.

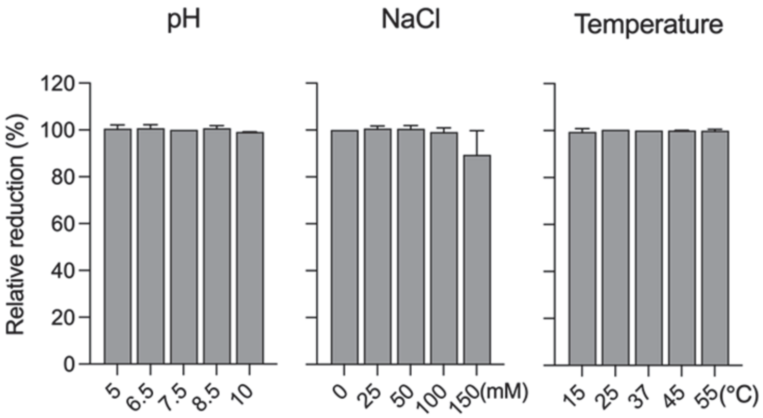

Figure S4. Effect of pH, NaCl, temperature on the antibacterial activity of CecA::ST01. CFU reduction assay was performed with 0.0625 μM of CecA::ST01 using freshly grown *A. baumannii* ATCC 17978 cells in the buffer containing different concentration of NaCl. To determine pH, temperature stability, CecA::ST01 was pre-incubated in the buffer with different pH or at different temperature for 1 h then used for the assay. Relative activity (%) was calculated by comparing the lytic activity under specific condition with the maximal activity (20 mM Tris-HCl, pH 7.5 at 37□).
